# Increased rate of complications in myasthenia gravis patients following hip and knee arthroplasty: a nationwide database study in the PearlDiver Database on 257,707 patients

**DOI:** 10.1080/17453674.2020.1865031

**Published:** 2021-01-04

**Authors:** William F Sherman, Victor J Wu, Sione A Ofa, Bailey J Ross, Ian D Savage-Elliott, Fernando L Sanchez

**Affiliations:** Department of Orthopaedic Surgery, Tulane University School of Medicine, New Orleans, LA, USA

## Abstract

Background and purpose — The increasing prevalence of total hip arthroplasty (THA) and total knee arthroplasty (TKA) within the growing elderly population is translating into a larger number of patients with neuromuscular conditions such as myasthenia gravis (MG) receiving arthroplasty. We compared systemic and joint complications following a THA or TKA between patients with MG and patients without MG.

Patients and methods — Patient records were queried from PearlDiver (Pearl Diver Inc, Fort Wayne, IN, USA), an administrative claims database, using ICD-9/ICD-10 and Current Procedural Terminology codes. In-hospital and 90-day post-discharge rates of systemic and joint complications were compared between the 2 cohorts.

Results — 372 patients with MG and 249,428 patients without MG who received a THA or TKA were included in the study. At 90 days post-discharge, MG patients exhibited exhibited between 1.6 and 15% higher rates of systemic complications, including cerebrovascular event, pneumonia, respiratory failure, sepsis, myocardial infarction, acute renal failure, anemia, and deep vein thrombosis (all p < 0.001). The same results were also found during the in-hospital time period. 90-day incidence of aseptic loosening was the only joint complication with significantly increased odds risk for the MG cohort (OR 5; 95% CI 2–12).

Interpretation — Patients with MG exhibited significantly higher risk for multiple systemic complications during the index hospital stay and in the acute post-discharge setting.

Myasthenia gravis (MG) is an autoimmune neuromuscular disease that causes fluctuating muscle weakness throughout the body (Ciafaloni 2019). The estimated global incidence and prevalence of MG between 1950 and 2007 was between 5 and 77 per 10^5^ persons respectively, and mortality was 0.1–0.9 per 10^5^ persons (Carr et al. 2010). Thymectomy improves clinical outcomes, reduces the need for immunosuppressive therapy, and may be curative in a subset of patients (Wolfe et al. 2016, Remes-Troche et al. 2002). Advancements in the treatment have improved life expectancy and increased the demand for arthroplasty in these patient (Carr et al. 2010, Cichos et al. 2019). As the demand for primary hip and knee arthroplasty is projected to increase (Sloan et al. 2018), it is imperative that surgeons have access to data outlining clinical outcomes following arthroplasty in MG patients.

Recent studies examining total joint replacement in patients with neuromuscular disorders have demonstrated increased rates of both prosthetic joint and systemic medical complications (Cichos et al. 2019). However, there remains a lack of research specifically aimed at identifying the risks and complications seen with arthroplasties in patients with MG. Prior studies examining postoperative complications in patients with MG have demonstrated increased risks of adverse medical events including re-intubation (Cichos et al. 2019), increased surgical site bleeding, myasthenic crisis, pneumonia, and septicemia (Marulli et al. 2013).

This study compares rates of postoperative complications following total hip arthroplasty (THA) and total knee arthroplasty (TKA) in MG vs. non-MG patients, with the intent of helping guide care teams in developing and implementing optimal surgical management plans when performing TJR on patients with MG.

## Patients and methods

This study used the Humana dataset, which contains the medical records of 25.4 million patients from 2007 to 2017 who were privately insured, commercially insured, or purchased their Medicare Advantage plans through Humana Health Insurance. Patient records were queried from PearlDiver (PearlDiver Inc, Fort Wayne, IN, USA), a commercially available administrative claims database, using ICD-9/ICD-10 and Current Procedural Terminology (CPT) codes.

A retrospective cohort design was used to compare patients who had MG and underwent a THA or TKA vs. patients without MG who underwent a THA or TKA. Patients who had undergone a THA or TKA were identified using both ICD and CPT codes. Exclusion criteria included patients receiving arthroplasty for pathologic or traumatic fractures. Inclusion criteria for patients with MG included a diagnosis of MG, made by board-certified physicians and defined by ICD diagnosis codes in the database, at any time before the THA or TKA. To exclude patients who no longer were at risk for symptomatic MG, patients with a prior history of thymectomy were also excluded. The ICD codes that defined the study groups are provided in Appendix Table A1 (see Supplementary data).

**Table 1.** Demographics and clinical characteristic comparisons. Values are count (%) unless otherwise specified

	THA/TKA		
	MG	no MG	
Demographic variable	(n = 372)	(n = 249,428)	p-value
Female sex	206 (55.4)	152,931 (61.3)	< 0.001
Age			
< 65	80 (21.5)	55,334 (22.2)	0.7
65–79	268 (72.0)	163,414 (65.5)	< 0.001
≥ 80	73 (19.6)	33,665 (13.5)	< 0.001
BMI **^a^**			
< 30	106 (38.7)	53,870 (34.9)	0.2
30–39	113 (41.2)	69,579 (45.1)	0.2
≥ 40	55 (20.1)	30,849 (20.0)	0.9
CCI, mean (SD)	3.0 (2.8)	1.9 (2.3)	<0.001
Specific comorbidities:			
Diabetes mellitus	217 (58.3)	104,304 (41.8)	< 0.001
Hypertension	332 (89.2)	199,152 (79.8)	< 0.001
COPD	152 (40.9)	68,945 (27.6)	< 0.001
Chronic kidney disease	0 (0)	287 (0.2)	NA
Congestive heart failure	64 (17.2)	24,965 (10.0)	< 0.001
Coronary artery disease	148 (39.8)	69,686 (27.9)	< 0.001
Rheumatoid arthritis	38 (10.2)	15,443 (6.2)	< 0.001
Tobacco use	84 (22.6)	47,351 (19.0)	0.1

**^a^** BMI data was available for 75% of MG and 62% of non-MG patients.

CCI, Charlson Comorbidity Index; COPD, chronic obstructive pulmonary disease; MG, myasthenia gravis; NA, not applicable; SD, standard deviation; THA, total hip arthroplasty; TKA, total knee arthroplasty.

Each cohort was queried for basic demographic information, clinical characteristics, and hospital course data such as age, sex, hospital region, BMI, length of stay (LOS), 90-day readmission rate, discharge status, Charlson comorbidity index (CCI), and comorbidities. Regional data was categorized based on the United States Census Bureau classification of Northeast, Midwest, South, and West. Discharge status codes (DC) were classified into home, nursing, expired, or discontinued against medical advice (DC-07). Home discharge included self-care (DC-01, 21) and home health (DC-06) codes. Expired discharge status included those with codes for expiration (DC-20, 40, 41). All other codes including discharge to or planned return to a short-term nursing facility, long-term care hospital, or other medical facility were classified as a nursing discharge status. Specific comorbidities were queried using ICD diagnosis codes from the database including the presence of diabetes mellitus, hypertension, chronic obstructive pulmonary disease, chronic kidney disease, congestive heart failure, coronary artery disease, rheumatoid arthritis, and tobacco use.

Incidences of postoperative systemic and joint complications were queried for the 2 patient cohorts. Both systemic and local joint complications were examined during the surgical encounter before discharge, and at 90 days post-discharge. Systemic complications queried included malignant hyperthermia, cerebrovascular event (stroke, non-traumatic hemorrhage, occlusion of cerebral arteries), anemia (post-hemorrhagic, iron deficiency from blood loss), acute renal failure, myocardial infarction (MI), pneumonia, sepsis, deep vein thrombosis (DVT), pulmonary embolism (PE), and respiratory failure. The codes used to define systemic complications are provided in Appendix Table A2 (see Supplementary data).

**Table 2.** Comparison of hospital region and course. Values are count (%) unless otherwise specified

	THA/TKA		
	MG	no MG	
Hospital course variable	(n = 372)	(n = 249,428)	p-value
Region			
South	230 (62)	143,403 (58)	0.1
Midwest	105 (28)	73,557 (30)	0.6
Northeast	5 (1.3)	6,426 (2.6)	0.1
West	32 (8.6)	26,186 (11)	0.2
Length-of-stay, mean (SD)	3.7 (3.0)	3.5 (4.4)	0.1
Discharge			
Home	290 (78)	162,656 (65)	< 0.001
Nursing	152 (41)	82,108 (33)	< 0.001
Expired	1 (0.3)	196 (0.08)	0.2
Against medical advice	0 (0)	66 (0.03)	NA
90-day readmission rate	50 (13)	32,307 (13)	0.7

For abbreviations, see Table 1.

Post-discharge joint complications queried included prosthetic joint infection (PJI), periprosthetic fracture, hip dislocation, and aseptic loosening. PJI was defined by procedural codes that indicated a surgical intervention for a deep joint infection to exclude superficial wound complications that would have been included in diagnosis codes for PJI. The codes used to define joint complications are provided in Appendix Table A3 (see Supplementary data)

**Table 3.** Comparison of systemic complications at 90 days postoperatively and during inpatient hospital stay. Values are count (%) and odds ratio (OR) with 95% confidence interval (CI)

	THA/TKA		
	MG	no MG	
Systemic complications	(n = 372)	(n = 249,428)	OR (95% CI)
Cerebrovascular event			
In-hospital	33 (8.9)	3,134 (1.3)	7.9 (5.4–11)
90-day	66 (18)	8,422 (3.4)	6.6 (4.9–8.7)
Pneumonia			
In-hospital	32 (8.6)	2,388 (1.0)	10 (6.8–14)
90-day	49 (13)	6,304 (2.5)	6.1 (4.5–8.3)
Respiratory failure			
In-hospital	30 (8.1)	3,310 (1.3)	6.7 (4.5–9.6)
90-day	37 (9.9)	5,175 (2.1)	5.5 (3.8–7.6)
Anemia			
In-hospital	120 (32.3)	72,094 (28.9)	1.2 (0.9–1.5)
90-day	102 (27.4)	39,005 (15.6)	2.1 (1.6–2.6)
Sepsis			
In-hospital	7 (1.9)	632 (0.3)	9.9 (4.7–18)
90-day	18 (4.8)	2,894 (1.2)	4.4 (2.6–6.9)
Acute MI			
In-hospital	11 (3.0)	1,019 (0.4)	7.4 (3.8–13)
90-day	11 (3.0)	2,283 (0.9)	3.3 (1.7–5.7)
Deep vein thrombosis			
In-hospital	13 (3.5)	2,689 (1.1)	3.3 (1.8–5.6)
90-day	33 (8.9)	12,331 (4.9)	1.9 (1.3–2.6)
Acute renal failure			
In-hospital	25 (6.7)	8,173 (3.3)	2.1 (1.4–3.2)
90-day	42 (11.3)	10,299 (4.1)	3.1 (2.2–4.2)
Pulmonary embolism			
In-hospital	14 (3.8)	1,559 (0.6)	6.2 (3.5–10)
90-day	21 (5.6)	4,260 (1.7)	3.4 (2.1–5.2)
Malignant hyperthermia:			
In-hospital	0 (0)	2 (< 0.1)	NA
90-day	0 (0)	2 (< 0.1)	NA

For abbreviations, see Table 1.

### Statistics

All data analyses were performed using the R statistical software (R Project for Statistical Computing, Vienna, Austria) integrated within PearlDiver with an α level set to 0.05. Multivariable logistic regression adjusting for patient sex, age, Charlson Comorbidity Index (CCI), and BMI was used to calculate odds ratios (OR) with corresponding 95% confidence intervals (CI) for rates of joint and systemic complications between the 2 cohorts. Proportions of various demographic and clinical characteristics in the MG and non-MG cohorts were compared using chi-square analysis to assess for baseline differences between the 2 patient populations. The null hypothesis was that different demographic proportions were the same between MG vs. non-MG cohorts. It is important to take into account the effect that age, sex, BMI, and CCI can have on outcomes of THA and TKA. Older patients experience higher risks of negative postoperative outcomes when undergoing either THA or TKA. In terms of in-hospital complications, examples of such poor outcomes include acute myocardial infarction, DVT or PE, surgical site infection, sepsis, hemorrhage, and mortality (Fang et al. 2015). Similarly, patients with a BMI classification of ≥ 30 are more likely to experience prosthetic failure and postoperative infection following TKA and THA (Boyce et al. 2019, Correa-Valderrama et al. 2019). For TKA, male patients have higher rates of revision surgery, mortality, hospital readmission, and wound infections compared with women, while female patients have increased risk of readmission, reoperation, and wound infection following THA (Singh et al. 2013, Patel et al. 2020). When evaluating comorbidity presence on mortality risk (CCI) for both THA and TKA, patients with a moderate (CCI score of 2) or high (CCI score of 3 or higher) comorbidity burden have a higher 90-day mortality risk in comparison with patients with a low comorbidity burden (CCI score of 1) (Glassou et al. 2017). The decision to control for age, sex, BMI, and CCI in the statistical analysis was because each variable has substantial potential for exerting confounding effects given the associated risks that each has on the outcomes following THA or TKA.

### Ethics, funding, and potential conflicts of interest

Institutional review board exemption by Tulane University Human Research Protection Program was granted for this study on June 11, 2020, because provided data was deidentified and compliant with the Health Insurance Portability and Accountability Act. This study received no funding or financial support from outside sources. Author FLS receives research support as a Principal Investigator from Medacta International (USA), and serves on the Content Committee for the American Academy of Orthopaedic Surgeons (AAOS) Hip and Knee. Author IDSE serves as a Co-Editor for Orthopedics Today Grand Rounds. All other authors declare no conflict of interests.

**Figure:**
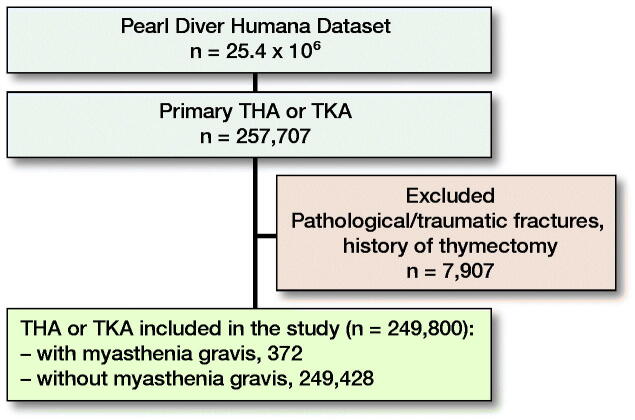
Patients included in this study. THA, total hip arthroplasty; TKA, total knee arthroplasty.

## Results

Between 2007 and 2017 in the PearlDiver database, 80,727 primary THAs and 176,980 primary TKAs were performed for a total of 257,707 arthroplasty procedures. After applying exclusion criteria, 249,428 patients who received THA or TKA did not have MG and 372 patients had MG (Figure). As outlined in Table 1, the MG cohort had a greater proportion of males (45% vs. 39%, p < 0.001) and older patients (age ≥ 80: 20% vs. 14%, p < 0.001). MG patients also had a higher average burden of comorbidities (CCI 3.0 vs. 1.9, p < 0.001). Additionally, MG patients had an increased mean LOS (3.7 days vs. 3.5 days, p = 0.1), and a higher 90-day readmission rate (13.4% vs. 12.9%, p = 0.7). Regarding disposition status, MG patients were more likely to discharge home (78% vs. 65%, p < 0.001) and to a nursing facility (41% vs. 33%, p < 0.001) (Table 2).

Systemic complications were more likely in patients with MG relative to non-MG patients in the 90-day post-discharge period (Table 3). Statistically significant findings included cerebrovascular event (OR 6.6), pneumonia (OR 6.1), respiratory failure (OR 5.5), sepsis (OR 4.4), pulmonary embolism (OR 3.4), myocardial infarction (OR 3.3), acute renal failure (OR 3.1), anemia (OR 2.1), and deep vein thrombosis (OR 1.9). Systemic complications were also more likely in the MG cohort during their inpatient hospital stay (Table 3). Complications that were statistically more likely for MG patients included pneumonia (OR 10), sepsis (OR 9.9), cerebrovascular event (OR 7.9), acute myocardial infarction (OR 7.4), respiratory failure (OR 6.7), and acute renal failure (OR 2.1).

With respect to joint-specific complications, almost no statistically significant differences were found between patients in the MG cohort and patients without MG for both the inpatient hospital stay duration and in the 90-day postoperative period (Table 4). The lone exception was aseptic loosening at 90 days post-discharge, which was found to be more likely in MG patients (90-day postop OR 5.1).

**Table 4.** 90 days postoperatively and during inpatient hospital stay comparison of joint-specific complications. Values are count (%) and odds ratio (OR) with 95% confidence interval (CI)

	THA/TKA		
	MG	no MG	
Local complications	(n = 372)	(n = 249,428)	OR (95% CI)
Prosthetic joint infection			
In-hospital	2 (0.5)	1,112 (0.5)	0.6 (0.1–1.6)
90-day	4 (1.1)	4,151 (1.7)	0.8 (0.3–1.8)
Periprosthetic fracture			
In-hospital	1 (0.3)	180 (0.7)	3.8 (0.2–17)
90-day	2 (0.5)	837 (0.3)	2.5 (0.6–6.5)
Aseptic loosening			
In-hospital	2 (0.5)	511 (0.2)	2.7 (0.4–8.4)
90-day	3 (0.8)	550 (0.2)	5.1 (1.6–12)
Prosthetic joint dislocation			
In-hospital	0 (0)	131 (< 0.1)	NA
90-day	0 (0)	353 (0.1)	NA

For abbreviations, see Table 1.

## Discussion

The majority of systemic complications analyzed were statistically significantly more likely and clinically relevant for MG patients relative to patients without MG. This held true for both in-hospital and at 90 days post-discharge. Despite the association of malignant hyperthermia with individuals with a known myopathy or neuromuscular disease (Wedel 1992) there was no occurrence of this complication in either cohort. It is possible the study was underpowered to discern a difference for this very rare complication. Additionally, malignant hyperthermia has a higher odds risk in children compared with adults (Rosenberg and Fletcher 1994, Rosenberg et al. 2015). Because the majority of patients undergoing total joint replacement are adults, low rates of malignant hyperthermia in this population are expected.

The increased occurrence of systemic complications in patients with MG aligns partially with the results of previous studies (Chang et al. 2017, Cichos et al. 2019). Similar to the findings by Chang et al. (2017), MG patients experienced higher rates of postoperative pneumonia and sepsis (Chang et al. 2017). Furthermore, these findings also support those by Cichos et al. (2019), which demonstrated higher rates of postoperative anemia and deep vein thrombosis in patients with MG (Cichos et al. 2019). Our study further expands the scope of systemic complications demonstrating MG patients are at a higher risk by also showing higher rates of cerebrovascular events, respiratory failure, acute MI, and acute renal failure. Chang et al. (2017) limited their examination of complications to 30 days post-discharge, and Cichos et al. (2019) limited their data collection to the perioperative event. Our data documented the increased occurrence of these complications during the hospital stay and within the 90-day post-discharge period. Given the similarities in findings to previous literature, our results suggest the presence of MG contributes to the increased odds risk for systemic complications after a TKA or THA.

In addition to the systemic complications, the data demonstrated MG patients were generally older (age 65–79, 72% vs. 66%, and age ≥ 80, 20% vs. 14%), had higher levels of non-obese classifications (BMI < 30, 39% vs. 5%), and had an increased burden of medical comorbidities (CCI 3.0 vs. 1.9) when compared with non-MG patients. Given the presence of comorbidities is associated with poorer outcomes after undergoing surgical operation (Misra et al. 2020), we used multivariable logistic regression to adjust for select high-impact confounding variables in an attempt to more accurately evaluate an MG as an independent risk factor in the increased occurrence of systemic complications.

The arthroplasty-specific complication found to be more likely for patients with MG was aseptic joint loosening within the 90-day post-discharge period (90-day postop OR 5.1; CI 1.6–12). The high odds ratio aligns with the findings by Cichos et al. (2019), who also demonstrated higher rates of aseptic loosening. However, the reliability of comparisons between our study and prior work is limited, given that previous studies examined joint complications in patients with any neuromuscular disorder undergoing total joint replacement where this study specifically examined total joint arthroplasty in patients with MG.

With TKA being moved to an outpatient designation by the Centers for Medicare and Medicaid Services (CMS), many total joint replacement procedures are being performed in surgery centers and centers without ICU capabilities. The increased systemic risks seen in this cohort of MG patients should guide surgeons who utilize outpatient surgery centers or centers without intensive care units to prepare for potential adverse events and account for the possibility of a systemic complication or need for sustained intubation postoperatively. The need for intubation of this cohort is a special consideration for resource management in the current COVID-19 pandemic. As elective cases are being reintroduced, arthroplasty in this vulnerable population should be closely examined to ensure appropriate support is available.

We acknowledge several limitations to this study. Given that a total knee replacement has a reported survival rate of 98% at 10 years postoperatively (Argenson et al. 2013, Jauregui et al. 2015, Brockett et al. 2018, Batailler et al. 2020), and a total hip replacement reported 97% survival rates at 10 years postoperatively (Kremers et al. 2012, Fowler et al. 2019, Chaudhry et al. 2020), by only measuring joint complications through the 90-day post-discharge period, this study is limited to only short-term data and excludes long-term complications. Another limitation is the lack of data within PearlDiver regarding the type of anesthesia used on MG patients during the arthroplasties; specifically, the codes used to indicate a spinal/epidural block did not delineate whether or not a patient also received general anesthesia, which may increase the rate of complications when compared with spinal anesthesia alone (Warren et al. 2020). With the complex nature of medical billing and lack of standardized coding for a variety of conditions, there is a possibility of coding bias with the manual entry of diagnosis/procedural codes. These errors are inherent with database-driven studies using administrative claims information. Furthermore, because this study includes patient data prior to and after 2015, the diagnosis/procedural codes do not match exactly across ICD-9 and ICD-10. Accuracy was ensured with the use of a code translator to match corresponding codes. Despite the utilization of a large database with a heterogeneous patient sample, the generalizability may be limited because the data is derived from one private insurance provider, which has a greater representation in the Midwestern and Southern regions of the United States. Although we used multivariable logistic regression to diminish the confounding effect of certain potential confounding variables, it is possible that not all relevant confounding variables were identified and controlled for. Additionally, with the inclusion of each variable into our logistic regression models, adjustment bias was introduced into the analysis. Lastly, since the PearlDiver database provides data only on patients who retained Humana health insurance during the time period queried, sampling bias is present.

Despite the regional limitation of the database used, the large size of this patient database allows for confidence in extrapolating the data to the general population. Additionally, this study is the first of its kind to examine in depth the complications seen specifically in myasthenia gravis patients when undergoing total joint replacement. Much of the prior literature has focused largely on complications seen in thymectomy operations, or on complications seen in patients with neuromuscular disorders, of which myasthenia gravis is its own subset of that group. Future research could further examine the relationship between total joint replacement and joint-specific complications for myasthenia gravis patients and other subgroups of neuromuscular disorders.

In conclusion, this study suggests patients with MG experience higher rates of systemic complications in both the acute postoperative period and 90-day post-discharge period. These findings have important clinical implications for both the patient and the surgeon. Moving forward, it is recommended that patients are counseled on the higher risk of the procedure and the risks associated with systemic complications. Surgeons should also be aware of the increased risks and take the appropriate preventative measures to minimize the systemic risks associated with a THA or TKA.

## Supplementary Material

Supplemental Material

## Supplementary data

Appendix Tables A1–A3 are available as supplementary data in the online version of this article, http://dx.doi.org/10.1080/17453674.2020.1865031
