# Supplementary material for: Increased rate of complications in myasthenia gravis patients following hip and knee arthroplasty: a nationwide database study in the PearlDiver Database on 257,707 patients
Source: Acta Orthop. 2021 Jan 4;92(2):176–81. doi: 10.1080/17453674.2020.1865031 (PMC8158199; doi:10.1080/17453674.2020.1865031)
Supplement: Supplemental Material [file IORT_A_1865031_SM0961.pdf]

Supplementary data

Table A1. Codes used for MG and THA/TKA and THA/TKA no MG cohorts

Primary THA codes		Primary TKA codes	Exclusion codes	
ICD-9-P-8151	ICD-10-P-0SRB04A	ICD-9-P-8154	ICD-9-D-82021	ICD-10-D-S72142B
ICD-10-P-0SR9019	ICD-10-P-0SRB04Z	ICD-10-P-0SRC07Z	ICD-9-D-82020	ICD-10-D-S72142C
ICD-10-P-0SR901A	ICD-10-P-0SRB07Z	ICD-10-P-0SRC0J9	ICD-9-D-8209	ICD-10-D-S72143B
ICD-10-P-0SR901Z	ICD-10-P-0SRB0J9	ICD-10-P-0SRC0JA	ICD-9-D-82031	ICD-10-D-S72143C
ICD-10-P-0SR9029	ICD-10-P-0SRB0JA	ICD-10-P-0SRC0JZ	ICD-9-D-82013	ICD-10-D-S72144B
ICD-10-P-0SR902A	ICD-10-P-0SRB0JZ	ICD-10-P0SRC0KZ	ICD-9-D-82030	ICD-10-D-S72144C
ICD-10-P-0SR902Z	ICD-10-P-0SRB0KZ	ICD-10-P-0SRC0L9	ICD-9-D-82010	ICD-10-D-S72145B
ICD-10-P-0SR9039	ICD-10-P-0SRE009	ICD-10-P-0SRC0LZ	ICD-9-D-82019	ICD-10-D-S72146B
ICD-10-P-0SR903A	ICD-10-P-0SRE00A	ICD-10-P-0SRD0J9	ICD-9-D-82012	ICD-10-D-S72146C
ICD-10-P-0SR903Z	ICD-10-P-0SRE00Z	ICD-10-P-0SRD0JA	ICD-9-D-82032	ICD-10-D-S72051A
ICD-10-P-0SR9049	ICD-10-P-0SRE019	ICD-10-P-0SRD0JZ	ICD-9-D-82011	ICD-10-D-S72052A
ICD-10-P-0SR904A	ICD-10-P-0SRE01A	ICD-10-P-0SRD0KZ	ICD-9-P-0780	ICD-10-D-S72061A
ICD-10-P-0SR904Z	ICD-10-P-0SRE01Z	ICD-10-P-0SRD0L9	ICD-10-P-07BM0ZX	ICD-10-D-S72062A
ICD-10-P-0SR907Z	ICD-10-P-0SRE039	ICD-10-P-0SRD0LZ	ICD-10-P-07BM0ZZ	ICD-10-D-S72059A
ICD-10-P-0SR90J9	ICD-10-P-0SRE03A	ICD-10-P-0SRT0J9	ICD-10-P-07BM3ZZ	ICD-10-D-S72065A
ICD-10-P-0SR90JA	ICD-10-P-0SRE03Z	ICD-10-P-0SRT0JA	ICD-10-P-07BM4ZX	ICD-10-D-S72064A
ICD-10-P-0SR90JZ	ICD-10-P-0SRE0J9	ICD-10-P-0SRT0JZ	ICD-10-P-07BM4ZZ	ICD-10-D-S72051A
ICD-10-P-0SR90KZ	ICD-10-P-0SRE0JA	ICD-10-P-0SRU0J9	ICD-10-D-S72001B	ICD-10-D-S72052B
ICD-10-P-0SRA009	ICD-10-P-0SRE0JZ	ICD-10-P-0SRU0JA	ICD-10-D-S72002B	ICD-10-D-S72061B
ICD-10-P-0SRA00A	ICD-10-P-0SRR019	ICD-10-P-0SRU0JZ	ICD-10-D-S72009B	ICD-10-D-S72063A
ICD-10-P-0SRA00Z	ICD-10-P-0SRR01A	ICD-10-P-0SRU0KZ	ICD-10-D-S72002C	ICD-10-D-S72064B
ICD-10-P-0SRA019	ICD-10-P-0SRR01Z	ICD-10-P-0SRV0J9	ICD-10-D-S72001C	ICD-10-D-S72066A
ICD-10-P-0SRA01A	ICD-10-P-0SRR039	ICD-10-P-0SRV0JA	ICD-10-D-S72009C	ICD-10-D-S72011B
ICD-10-P-0SRA01Z	ICD-10-P-0SRR03A	ICD-10-P-0SRV0JZ	ICD-10-D-S72041B	ICD-10-D-S72012B
ICD-10-P-0SRA039	ICD-10-P-0SRR03Z	ICD-10-P-0SRW0J9	ICD-10-D-S72042B	ICD-10-D-S72012C
ICD-10-P-0SRA03A	ICD-10-P-0SRR07Z	ICD-10-P-0SRW0JA	ICD-10-D-S72043B	ICD-10-D-S72019B
ICD-10-P-0SRA03Z	ICD-10-P-0SRR0J9	ICD-10-P-0SRW0JZ	ICD-10-D-S72043C	
ICD-10-P-0SRA07Z	ICD-10-P-0SRR0JA	ICD-10-P-0SRW0KZ	ICD-10-D-S72044B	
ICD-10-P-0SRA0J9	ICD-10-P-0SRR0JZ	CPT-27440	ICD-10-D-S72046B	
ICD-10-P-0SRA0JA	ICD-10-P-0SRR0KZ	CPT-27441	ICD-10-D-S72091B	
ICD-10-P-0SRA0JZ	ICD-10-P-0SRS019	CPT-27442	ICD-10-D-S72091C	
ICD-10-P-0SRA0KZ	ICD-10-P-0SRS01A	CPT-27443	ICD-10-D-S72092B	
ICD-10-P-0SRB019	ICD-10-P-0SRS01Z	CPT-27445	ICD-10-D-S72101B	
ICD-10-P-0SRB01A	ICD-10-P-0SRS039	CPT-27446	ICD-10-D-S72101C	
ICD-10-P-0SRB01Z	ICD-10-P-0SRS03A	CPT-27447	ICD-10-D-S72101E	
ICD-10-P-0SRB029	ICD-10-P-0SRS03Z		ICD-10-D-S72101F	
ICD-10-P-0SRB02A	ICD-10-P-0SRS0J9		ICD-10-D-S72101J	
ICD-10-P-0SRB02Z	ICD-10-P-0SRS0JA	Myasthenia gravis codes	ICD-10-D-S72102B	
ICD-10-P-0SRB039	ICD-10-P-0SRS0JZ	ICD-9-D-35800	ICD-10-D-S72102C	
ICD-10-P-0SRB03A	ICD-10-P-0SRS0KZ	ICD-10-D-G7000	ICD-10-D-S72109B	
ICD-10-P-0SRB03Z	CPT-27130		ICD-10-D-S72141B	
ICD-10-P-0SRB049			ICD-10-D-S72141C	

MG, myasthenia gravis; THA, total hip arthroplasty; TKA, total knee arthroplasty; ICD-9/ICD-10, International Classification of Diseases, Ninth Revision and Tenth Revision.

Appendix Table A2. Codes used to evaluate for systemic complications

- Acute renal failure
  - ICD-9-D-5845
  - ICD-9-D-5846
  - ICD-9-D-5847
  - ICD-9-D-5848
  - ICD-9-D-5849
  - ICD-9-D-5800
  - ICD-9-D-5804
  - ICD-9-D-58081
  - ICD-9-D-58089
  - ICD-9-D-5809
  - ICD-10-D-N170
  - ICD-10-D-N171
  - ICD-10-D-N172
  - ICD-10-D-N178
  - ICD-10-D-N179
  - ICD-10-D-N19
  - ICD-10-D-N990
  - ICD-10-D-N000
  - ICD-10-D-N001
  - ICD-10-D-N002
  - ICD-10-D-N003
  - ICD-10-D-N004
  - ICD-10-D-N005
  - ICD-10-D-N006
  - ICD-10-D-N007
  - ICD-10-D-N008
  - ICD-10-D-N009
- Anemia
  - ICD-9-D-2851
  - ICD-9-D-2800
  - ICD-10-D-D500
  - ICD-10-D-D62
- Cerebrovascular event
  - ICD-9-D-430
  - ICD-9-D-431
  - ICD-9-D-4320
  - ICD-9-D-4321
  - ICD-9-D-4329
  - ICD-9-D-43300
  - ICD-9-D-43301
  - ICD-9-D-43310
  - ICD-9-D-43311
  - ICD-9-D-43320
  - ICD-9-D-43321
  - ICD-9-D-43330
  - ICD-9-D-43331
  - ICD-9-D-43380
  - ICD-9-D-43381
  - ICD-9-D-43390
  - ICD-9-D-43391
  - ICD-9-D-43400
  - ICD-9-D-43401
  - ICD-9-D-43410
  - ICD-9-D-43411
  - ICD-9-D-43490
  - ICD-9-D-43491
  - ICD-10-D-I6000
  - ICD-10-D-I6001
  - ICD-10-D-I6002
  - ICD-10-D-I6010
  - ICD-10-D-I6011
  - ICD-10-D-I6012
  - ICD-10-D-I602
  - ICD-10-D-I6020
  - ICD-10-D-I6021
  - ICD-10-D-I6022
  - ICD-10-D-I6030
  - ICD-10-D-I6031
  - ICD-10-D-I6032
  - ICD-10-D-I604
  - ICD-10-D-I6050
  - ICD-10-D-I6051
  - ICD-10-D-I6052
  - ICD-10-D-I606
  - ICD-10-D-I607
  - ICD-10-D-I608
  - ICD-10-D-I609
  - ICD-10-D-I610
  - ICD-10-D-I611
  - ICD-10-D-I612
  - ICD-10-D-I613
  - ICD-10-D-I614
  - ICD-10-D-I615
  - ICD-10-D-I616
  - ICD-10-D-I618
  - ICD-10-D-I619
  - ICD-10-D-I6200
  - ICD-10-D-I6201
  - ICD-10-D-I6202
  - ICD-10-D-I6203
  - ICD-10-D-I629
  - ICD-10-D-I6302
  - ICD-10-D-I6312
  - ICD-10-D-I6322
  - ICD-10-D-I651
  - ICD-10-D-I63031
  - ICD-10-D-I63032
  - ICD-10-D-I63033
  - ICD-10-D-I63039
  - ICD-10-D-I63131
  - ICD-10-D-I63132
  - ICD-10-D-I63133
  - ICD-10-D-I63139
  - ICD-10-D-I63231
  - ICD-10-D-I63232
  - ICD-10-D-I63233
  - ICD-10-D-I63239
  - ICD-10-D-I63011
  - ICD-10-D-I63012
  - ICD-10-D-I63013
  - ICD-10-D-I63019
  - ICD-10-D-I63111
  - ICD-10-D-I63112
  - ICD-10-D-I63113
  - ICD-10-D-I63119
  - ICD-10-D-I63211
  - ICD-10-D-I63212
  - ICD-10-D-I63213
  - ICD-10-D-I63219
  - ICD-10-D-I6300
  - ICD-10-D-I6309
  - ICD-10-D-I6310
  - ICD-10-D-I6319
  - ICD-10-D-I6320
  - ICD-10-D-I6329
  - ICD-10-D-I658
  - ICD-10-D-I659
  - ICD-10-D-I6501
  - ICD-10-D-I6502
  - ICD-10-D-I6503
  - ICD-10-D-I6509
  - ICD-10-D-I6521
  - ICD-10-D-I6522
  - ICD-10-D-I6523
  - ICD-10-D-I6529
  - ICD-10-D-G458
  - ICD-10-D-G459
  - ICD-10-D-I6330
  - ICD-10-D-I63311
  - ICD-10-D-I63312
  - ICD-10-D-I63313
  - ICD-10-D-I63319
  - ICD-10-D-I63321
  - ICD-10-D-I63322
  - ICD-10-D-I63323
  - ICD-10-D-I63329
  - ICD-10-D-I63331
  - ICD-10-D-I63332
  - ICD-10-D-I63333
  - ICD-10-D-I63339
  - ICD-10-D-I63341
  - ICD-10-D-I63342
  - ICD-10-D-I63343
  - ICD-10-D-I63349
  - ICD-10-D-I6339
  - ICD-10-D-I6340
  - ICD-10-D-I63411
  - ICD-10-D-I63412
  - ICD-10-D-I63413
  - ICD-10-D-I63419
  - ICD-10-D-I63421
  - ICD-10-D-I63422
  - ICD-10-D-I63423
  - ICD-10-D-I63429
  - ICD-10-D-I63431
  - ICD-10-D-I63432
  - ICD-10-D-I63433
  - ICD-10-D-I63439
  - ICD-10-D-I63441
  - ICD-10-D-I63442
  - ICD-10-D-I63443
  - ICD-10-D-I63449
  - ICD-10-D-I6349
  - ICD-10-D-I6350
  - ICD-10-D-I63511
  - ICD-10-D-I63512
  - ICD-10-D-I63513
  - ICD-10-D-I63519
  - ICD-10-D-I63521
  - ICD-10-D-I63522
  - ICD-10-D-I63523
  - ICD-10-D-I63529
  - ICD-10-D-I63531
  - ICD-10-D-I63532
  - ICD-10-D-I63533
  - ICD-10-D-I63539
  - ICD-10-D-I63541
  - ICD-10-D-I63542
  - ICD-10-D-I63543
  - ICD-10-D-I63549
  - ICD-10-D-I6359
  - ICD-10-D-I636
  - ICD-10-D-I638
  - ICD-10-D-I639
  - ICD-10-D-I6601
  - ICD-10-D-I6602
  - ICD-10-D-I6603
  - ICD-10-D-I6609
  - ICD-10-D-I6611
  - ICD-10-D-I6612
  - ICD-10-D-I6613
  - ICD-10-D-I6619
  - ICD-10-D-I6621
  - ICD-10-D-I6622
  - ICD-10-D-I6623
  - ICD-10-D-I6629
  - ICD-10-D-I668
  - ICD-10-D-I669
  - ICD-9-D-4359
  - ICD-9-D-4358
- Deep vein thrombosis
  - ICD-9-D-45340
  - ICD-9-D-45341
  - ICD-9-D-45342
  - ICD-9-D-45111
  - ICD-9-D-45119
  - ICD-9-D-45389
  - ICD-9-D-4539
  - ICD-9-D-4512
  - ICD-9-D-45350
  - ICD-9-D-45351
  - ICD-9-D-45352
  - ICD-10-D-I82401
  - ICD-10-D-I82402
  - ICD-10-D-I82403
  - ICD-10-D-I82409
  - ICD-10-D-I82491
  - ICD-10-D-I82492
  - ICD-10-D-I82493
  - ICD-10-D-I82499
  - ICD-10-D-I824Y1
  - ICD-10-D-I824Y2
  - ICD-10-D-I824Y3
  - ICD-10-D-I824Y9
  - ICD-10-D-I824Z1
  - ICD-10-D-I824Z2
  - ICD-10-D-I824Z3
  - ICD-10-D-I824Z9
  - ICD-10-D-I82501
  - ICD-10-D-I82502
  - ICD-10-D-I82503
  - ICD-10-D-I82509
  - ICD-10-D-I82591
  - ICD-10-D-I82592
  - ICD-10-D-I82593
  - ICD-10-D-I82599
  - ICD-10-D-I825Y1
  - ICD-10-D-I825Y2
  - ICD-10-D-I825Y3
  - ICD-10-D-I825Y9
  - ICD-10-D-I825Z1
  - ICD-10-D-I825Z2
  - ICD-10-D-I825Z3
  - ICD-10-D-I825Z9
- Malignant hyper-thermia
  - ICD-9-D-99586
  - ICD-10-D-T883XXA
  - ICD-10-D-T883XXD
  - ICD-10-D-T883XXS
- Myocardial infarction
  - ICD-9-D-41000
  - ICD-9-D-41001
  - ICD-9-D-41002
  - ICD-9-D-41010
  - ICD-9-D-41011
  - ICD-9-D-41012
  - ICD-9-D-41020
  - ICD-9-D-41021
  - ICD-9-D-41022
  - ICD-9-D-41030
  - ICD-9-D-41031
  - ICD-9-D-41032
  - ICD-9-D-41040
  - ICD-9-D-41041
  - ICD-9-D-41042
  - ICD-9-D-41050
  - ICD-9-D-41051
  - ICD-9-D-41052
  - ICD-9-D-41080
  - ICD-9-D-41081
  - ICD-9-D-41082
  - ICD-9-D-41090
  - ICD-9-D-41091
  - ICD-9-D-41092
  - ICD-9-D-41070
  - ICD-9-D-41071
  - ICD-9-D-41072
  - ICD-9-D-41060
  - ICD-9-D-41061
  - ICD-9-D-41062
  - ICD-10-D-I214
  - ICD-10-D-I213
  - ICD-10-D-I2119
  - ICD-10-D-I2109
  - ICD-10-D-I2129
  - ICD-10-D-I240
  - ICD-10-D-I2111
  - ICD-10-D-I2102
  - ICD-10-D-I222
  - ICD-10-D-I2121
  - ICD-10-D-I229
  - ICD-10-D-I2101
  - ICD-10-D-I221
  - ICD-10-D-I220
  - ICD-10-D-I228
- Pneumonia
  - ICD-9-D-413
  - ICD-9-D-4800
  - ICD-9-D-4801
  - ICD-9-D-4802
  - ICD-9-D-4803
  - ICD-9-D-4808
  - ICD-9-D-4809
  - ICD-9-D-481
  - ICD-9-D-4820
  - ICD-9-D-4821
  - ICD-9-D-4822
  - ICD-9-D-48230
  - ICD-9-D-48231
  - ICD-9-D-48232
  - ICD-9-D-48239
  - ICD-9-D-48240
  - ICD-9-D-48241
  - ICD-9-D-48242
  - ICD-9-D-48249
  - ICD-9-D-48281
  - ICD-9-D-48282
  - ICD-9-D-48283
  - ICD-9-D-48284
  - ICD-9-D-48289
  - ICD-9-D-4829
  - ICD-9-D-4830
  - ICD-9-D-4831
  - ICD-9-D-4838
  - ICD-9-D-4841
  - ICD-9-D-485
  - ICD-9-D-486
  - ICD-9-D-4870
  - ICD-9-D-99731
  - ICD-9-D-99732
  - ICD-10-D-J189
  - ICD-10-D-J188
  - ICD-10-D-J180
  - ICD-10-D-J151
  - ICD-10-D-J157
  - ICD-10-D-J150
  - ICD-10-D-J1289
  - ICD-10-D-J09X1
  - ICD-10-D-J851
  - ICD-10-D-J1001
  - ICD-10-D-J1108
  - ICD-10-D-J153
  - ICD-10-D-J122
  - ICD-10-D-J1281
- Pulmonary embolism
  - ICD-9-D-41511
  - ICD-9-D-41513
  - ICD-9-D-41519
  - ICD-9-D-4162
  - ICD-10-D-I2609
  - ICD-10-D-I2699
  - ICD-10-D-I2782
- Respiratory failure
  - ICD-9-D-51853
  - ICD-9-D-51851
  - ICD-9-D-51883
  - ICD-9-D-51884
  - ICD-9-D-51881
  - ICD-9-D-51852
  - ICD-9-D-51882
  - ICD-10-D-J9601
  - ICD-10-D-J9600
  - ICD-10-D-J9690
  - ICD-10-D-J9621
  - ICD-10-D-J9610
  - ICD-10-D-J9611
  - ICD-10-D-J9602
  - ICD-10-D-J9620
  - ICD-10-D-J9622
  - ICD-10-D-J9691
  - ICD-10-D-J95821
  - ICD-10-D-J9612
  - ICD-10-D-J9692
  - ICD-10-D-J95822
  - ICD-10-D-J952
  - ICD-10-D-J953
- Sepsis
  - ICD-9-D-0031
  - ICD-9-D-0223
  - ICD-9-D-0380
  - ICD-9-D-03810
  - ICD-9-D-03811
  - ICD-9-D-03812
  - ICD-9-D-03819
  - ICD-9-D-0382
  - ICD-9-D-0383
  - ICD-9-D-03840
  - ICD-9-D-03841
  - ICD-9-D-03842
  - ICD-9-D-03843
  - ICD-9-D-03844
  - ICD-9-D-03849
  - ICD-9-D-0388
  - ICD-9-D-0389
  - ICD-9-D-0545
  - ICD-9-D-223
  - ICD-9-D-380
  - ICD-9-D-3812
  - ICD-9-D-382
  - ICD-9-D-383
  - ICD-9-D-3840
  - ICD-9-D-3842
  - ICD-9-D-3843
  - ICD-9-D-3844
  - ICD-9-D-388
  - ICD-9-D-389
  - ICD-9-D-545
  - ICD-10-D-A021
  - ICD-10-D-A227
  - ICD-10-D-A267
  - ICD-10-D-A327
  - ICD-10-D-A400
  - ICD-10-D-A401
  - ICD-10-D-A403
  - ICD-10-D-A408
  - ICD-10-D-A409
  - ICD-10-D-A4101
  - ICD-10-D-A4102
  - ICD-10-D-A411
  - ICD-10-D-A412
  - ICD-10-D-A413
  - ICD-10-D-A414
  - ICD-10-D-A4150
  - ICD-10-D-A4151
  - ICD-10-D-A4152
  - ICD-10-D-A4153
  - ICD-10-D-A4159
  - ICD-10-D-A4181
  - ICD-10-D-A4189
  - ICD-10-D-A419
  - ICD-10-D-A427
  - ICD-10-D-A5486
  - ICD-10-D-B377
  - ICD-10-D-R6520
  - ICD-10-D-R6521

ICD-9/ICD-10, International Classification of Disease, Ninth Revision and Tenth Revision.

Appendix Table A3. Codes used to evaluate for joint complications

Joint infection	Periprosthetic fracture	Aseptic loosening	Prosthetic dislocation	
ICD-9-D-99667	ICD-9-D-99644	ICD-9-D-99641	ICD-9-P-7975	ICD-10-P-0SSB44Z
ICD-9-D-99666	ICD-9-D-99644	ICD-9-D-99641	ICD-9-P-7985	ICD-10-P-0SSBX4Z
ICD-10-D-T8451XA	ICD-10-D-M9701XA	ICD-10-D-T84030A	ICD-9-P-7976	ICD-10-P-0SSBX5Z
ICD-10-D-T8451XD	ICD-10-D-M9702XA	ICD-10-D-T84030D	ICD-9-P-7986	ICD-10-P-0SSBXZZ
ICD-10-D-T8451XS	ICD-10-D-M9711XA	ICD-10-D-T84030S	ICD-10-P-0SS904Z	ICD-10-P-0SSC04Z
ICD-10-D-T8452XA	ICD-10-D-M9711XD	ICD-10-D-T84031A	ICD-10-P-0SS905Z	ICD-10-P-0SSC0ZZ
ICD-10-D-T8452XD	ICD-10-D-M9711XS I	ICD-10-D-T84031D	ICD-10-P-0SS90ZZ	ICD-10-P-0SSC3ZZ
ICD-10-D-T8452XS	CD-10-D-M9712XA	ICD-10-D-T84031S	ICD-10-P-0SS934Z	ICD-10-P-0SSC4ZZ
ICD-10-D-T8453XA	ICD-10-D-M9712XD	ICD-10-D-T84032A	ICD-10-P-0SS93ZZ	ICD-10-P-0SSCXZZ
ICD-10-D-T8453XD	ICD-10-D-M9712XS	ICD-10-D-T84032D	ICD-10-P-0SS944Z	ICD-10-P-0SSD04Z
ICD-10-D-T8453XS	ICD-10-D-T84042A	ICD-10-D-T84032S	ICD-10-P-0SS9X4Z	ICD-10-P-0SSD0ZZ
ICD-10-D-T8454XA	ICD-10-D-T84042D	ICD-10-D-T84033A	ICD-10-P-0SS9XZZ	ICD-10-P-0SSDX5Z
ICD-10-D-T8454XD	ICD-10-D-T84042S	ICD-10-D-T84033D	ICD-10-P-0SSB04Z	ICD-10-P-0SSDXZZ
ICD-10-D-T8454XS	ICD-10-D-T84043A	ICD-10-D-T84033S	ICD-10-P-0SSB0ZZ	
	ICD-10-D-T84043D		ICD-10-P-0SSB34Z	
	ICD-10-D-T84043S		ICD-10-P-0SSB3ZZ	

ICD-9/10, International Classification of Diseases, Ninth Revision and Tenth Revision.
